# Construction of a Seasonal Difference-Geographically and Temporally Weighted Regression (SD-GTWR) Model and Comparative Analysis with GWR-Based Models for Hemorrhagic Fever with Renal Syndrome (HFRS) in Hubei Province (China)

**DOI:** 10.3390/ijerph13111062

**Published:** 2016-10-29

**Authors:** Liang Ge, Youlin Zhao, Zhongjie Sheng, Ning Wang, Kui Zhou, Xiangming Mu, Liqiang Guo, Teng Wang, Zhanqiu Yang, Xixiang Huo

**Affiliations:** 1 State Key Laboratory of Information Engineering in Surveying, Mapping and Remote Sensing, Wuhan University, Wuhan 430079, China; 2 Tianjin Institute of Surveying and Mapping, Tianjin 300381, China; geliang0022@126.com (Z.S.); tjzk2@sina.com (K.Z.); jon1117@sina.com (L.G.); 3 Business School of Hohai University, Nanjing 211100, China; sobzyl@hhu.edu.cn (Y.Z.); tengw8837@hhu.edu.cn (T.W.); 4 First Crust Deformation Monitoring and Application Center, China Earthquake Administration, Tianjin 300180, China; kingning3344@126.com; 5 School of Information Studies of University of Wisconsin-Milwaukee, Milwaukee, WI 53211, USA; mux@uwm.edu; 6 State Key Laboratory of Virology, Institute of Medical Virology, School of Medicine, Wuhan University, Wuhan 430079, China; geliang0021@163.com; 7 Hubei Provincial Center for Disease Control and Prevention, Wuhan 430079, China; xixianghuo@163.com

**Keywords:** HFRS, GWR-based models, GTWR, SD-GTWR, spatiotemporal pattern

## Abstract

Hemorrhagic fever with renal syndrome (HFRS) is considered a globally distributed infectious disease which results in many deaths annually in Hubei Province, China. In order to conduct a better analysis and accurately predict HFRS incidence in Hubei Province, a new model named Seasonal Difference-Geographically and Temporally Weighted Regression (SD-GTWR) was constructed. The SD-GTWR model, which integrates the analysis and relationship of seasonal difference, spatial and temporal characteristics of HFRS (HFRS was characterized by spatiotemporal heterogeneity and it is seasonally distributed), was designed to illustrate the latent relationships between the spatio-temporal pattern of the HFRS epidemic and its influencing factors. Experiments from the study demonstrated that SD-GTWR model is superior to traditional models such as GWR- based models in terms of the efficiency and the ability of providing influencing factor analysis.

## 1. Introduction

HFRS is a zoonosis caused by different species of hantavirus, such as Hantaan virus (HTNV) and Seoul virus (SEOV) [1]. As an extremely dangerous viral disease, HFRS is a characterized by fever, acute renal dysfunction and haemostatic manifestations [2] and globally it causes a considerable number of deaths each year [3]. China accounted for approximately 90% of all HFRS cases in the world from 1990 to 2003 [4]. From 1981 to 2005, more than 20,000 cases of HFRS occurred annually, and in 1986, it caused 2569 deaths [5]. Hubei Province is one of the main HFRS outbreak areas in mainland China since the first case was discovered in Wuhan, China, in the 1980s. The fatality rate of HFRS in Hubei Province was 8.31 per 100,000 in 1980, which was the record until the early 21st century [6]. From 2004 to 2014, the average annual HFRS incidence rate in Hubei Province dropped to 0.43 per 100,000 and the average annual HFRS fatality rate dropped to 0.01 per 100,000 (the statistical data was acquired from the Hubei Provincial Center for Disease Control and Prevention and Chinese Center for Disease Control and Prevention). Zhang et al., found that from 1980 to 2009, the annual average incidence of the HFRS in Hubei Province in the countryside area ranged from 0 to 655.04 per 100,000. The highest incidence rate, which exceeded 40 per 100,000, happened in Hubei Province in 1983 [6]. Finding the reasons and key factors that contribute to HFRS outbreaks could assist their prediction and control. Understanding the spatial and temporal heterogeneity of HFRS is very important for designing and implementing effective control of HFRS epidemics [7,8].

Recent studies have used varieties of methods to discover the spatial and temporal variations of HFRS outbreak patterns in different locations of China over the past thirty years [9]. For instance, the Trend Surface Analysis (TSA) method was adopted to identify spreading tendency of HFRS in Shandong Province, China, from 1973 to 2005. The result demonstrated that the transmission pattern of HFRS shifted over time [10]. Local Indicators of Spatial Association and Kulldorff’s space-time scan statistic were used by Zhang et al., [11] to detect local high-risk space-time clusters of HFRS in China from 2005 to 2012. Autocorrelation characteristics were determined and the spatiotemporal dynamics of HFRS transmission were examined in their research.

After figuring out the spatial and temporal distribution pattern of HFRS, studying the factors influencing HFRS would be helpful for the control of HFRS outbreaks. In addition, meteorological factors and natural environmental factors are also key factors as discovered by Thomson et al., [12], Zhang et al., [13], and Bi et al., [14].

Meteorological factors might play an important role in the transmission of HFRS [15]. Cross-correlation and autocorrelation analysis were performed to detect the lagged effect of climate factors in Shenyang City (Liaoning Province, China) from 2004 to 2009. It was concluded that the transmission of HFRS was associated with local temperature, relative humidity, rainfall, air pressure, and wind velocity [16]. Granger causality (G-causality) tests were performed to measure the correlation coefficients between influencing factors (temperature, relative humidity, and rainfall) and the number of HFRS cases. It was demonstrated that the variations in HFRS incidence were significantly associated with local precipitation, humidity, and temperature [10].

Like the meteorological factors, natural environmental factors were also found to be the basis for the transmission pattern of HFRS. Spatial correlation analysis was used to detect the influencing factors of HFRS in Jiangsu Province. Besides the climate factors, the population density of humans and rodents also had major impacts on outbreaks of HFRS [17]. In another study, multivariate logistic regression analysis was conducted to explore the spatial distribution of hantavirus infections and their environmental influencing factors [18]. The results indicated that HFRS infections were significantly associated with rice agriculture, average surface elevation and rodent population density, with rodent population density being the most influential factor.

As commonly accepted regression models, Ordinary Least Squares (OLS)—and Geographically Weighted Regression (GWR)—based models were adopted to conduct correlation analysis or regression analysis for HFRS. For instance, the OLS method was used to discover the relationships among climate variables, density of mice, autumn crop production and incidence of HFRS in China [14]. A spatial analysis model (GWR model) was used to verify the geographical aspects of the HIV/AIDS epidemic in Japan [19]. Based on a GWR model, Huang developed in 2010 a new method named Geographically and Temporally Weighted Regression (GTWR) to process data characterized by both geographical and temporal information [20].

The main purpose of this study is to identify the key factors that influence the outbreaks of HFRS and to explore how these factors are associated with the spatiotemporal pattern of HFRS outbreaks. In this study, the number of HFRS cases is regarded as the dependent variable and the influencing factors are independent variables. Regression analysis is a powerful tool for detecting relationships between the dependent variable and independent variables [21,22].

As we mentioned before, HFRS is a specifically spatial distributed epidemic. Each model (OLS, GWR and GTWR) has their own merits and limitations to verify the spatial-temporal characteristics of HFRS.

Firstly, OLS is a traditional statistical method that uses least squares in linear regression analysis. Its weighting calculation neglects the spatial location information. The spatial heterogeneity cannot be integrated with OLS in the analysis of HFRS.

Secondly, both of temporal information and spatial information should be considered for HFRS. GWR model uses spatial weights to evaluate the connections between the dependent variable and independent variables [21], while ignoring temporal information.

Thirdly, GTWR model employs both non-stationary spatial and temporal weights matrices to represent spillovers from neighboring geographical locations, which improves the accuracy of estimation compared with the GWR model. HFRS caused a strong seasonal epidemic in Changsha City (Hunan Province) from 2000 to 2009 and in Anhui Province [23,24]. In our previous study, we have proved that the seasonal epidemic pattern of HFRS in Hubei Province was characterized by a shift from the unimodal type (autumn/winter peak) to the bimodal type [6]. OLS models and GWR- based models (GWR and GTWR) cannot deal with the unique seasonal characteristics of HFRS.

Considering the seasonal characteristics of HFRS, a new Seasonal Difference-Geographically and Temporally Weighted Regression model (SD-GTWR) was developed in this research. This SD-GTWR model was developed based on the GTWR model. As a result, it inherited the spatial and temporal characteristics of the GTWR model and thus can simulate the tendencies of HFRS epidemics with consideration of both their spatial and temporal variations. However, the SD-GTWR model integrates the seasonally distributed characteristics of HFRS epidemics and has a capability to perform seasonal difference calculations to eliminate the temporal non-stationary problem of HFRS data. Specifically, the new SD-GTWR model displays three characteristics as follows:

First, evolved from traditional linear regression methods (such as OLS), the GWR model estimated regression models based on spatial non-stationarity analysis [21]. Spatial variations can be measured by a specified neighborhood value for each location data in the GWR model [25]. Temporal parameters were considered when the data showed both spatial and temporal characteristics. As a result, GTWR is considered a spatiotemporal non-stationary model. Spatial and temporal weighted matrixes are inherited from GTWR.

Secondly, in the SD-GTWR model, seasonal difference calculations can eliminate the impact of seasonal variation and the temporal non-stationarity of HFRS case data. Seasonal variation is an important characteristic of HFRS outbreaks in every province of mainland China [6,23,24]. Seasonal fluctuations in temporal dimension have special effects on the accuracy of coefficient analysis on factors.

Thirdly, SD-GTWR utilized Incremental Spatial Autocorrelation (ISA) to verify the initial bandwidth value which is an important parameter during the iteration calculation of the model. When big data is utilized an enormous amount of time will be consumed for the determination of bandwidth values due to the numerous iterations required in the calculations. ISA provides a new way to reduce the computation time. Although the matrix order has thousands of levels, bandwidth selection range will be narrowed significantly with SD-GTWR model.

## 2. Construction of the Seasonal Difference-Geographically and Temporally Weighted Regression Model (SD-GTWR)

Extended from GTWR model, the SD-GTWR model not only inherits the functions of GTWR, but also has its own particular advantages.

### 2.1. Principle of the Geographically and Temporally Weighted Regression Model (GTWR)

When making GWR estimations, there are commonly three types of models that could be selected. These are the Gaussian GWR model (GWGR), Poisson GWR model (GWPR) and the Logistic GWR model (GWLR). 

The GWGR model is simple and commonly used in the related studies about this issue [26]. In order to compare our result to similar studies, and also for convenience consideration, we decided to use GWGR as the basis for our new SD-GTWR model. The GWGR model uses spatial weight functions and bandwidths to avoid getting a negative estimation result. Meanwhile, the amount of data used in this study is large enough to serve GWGR model estimations.

From the previous research [20], GTWR was expressed as:

(1) $$y_{i}=\beta_{0}\left(u_{i},v_{i},t_{i}\right)+\sum_{k=1}^{d}\beta_{k}\left(u_{i},v_{i},t_{i}\right)x_{ik}+\varepsilon_{i},i=1,2,...,n$$

where *u_i_* represents the x coordinate of observed location *i, v_i_* represents the y coordinate of the observed location *i*, and then *t_i_* represents the observed time for the observed location *i*. *y_i_*, the dependent variable of this model, represents the number of HFRS cases at location (*u_i_*,*v_i_*,*t_i_*). *x_ik_* is the corresponding possible influencing factor. d represents the number of categories of influencing factors, and n represents the count number of observed locations. The parameter *β_k_*(*u_i_*,*v_i_*,*t_i_*), *k* = 0,1,2,…,*d*) is an arbitrary function for the observed location (*u_i_*,*v_i_*,*t_i_*), and it is an unknown parameter for the observed location (*u_i_*,*v_i_*,*t_i_*). ε*_i_* is a constant, which represents the random error for the observed location *i*. 

This model is different from other “fixed” coefficient estimation models, such as OLS or the Autoregressive Integrated Moving Average (ARIMA) model. It allows the parameter estimates to vary across space and time to capture the local effects in different time. Spatial autocorrelation hypothesis assumes that observed data closed to observed location *i* have greater impacts than the data spatially far from observed location *i* does. From Equation (1), parameter estimation for (*u_i_*,*v_i_*, *t_i_*) can be expressed as:

(2) $$\hat{\beta }\left(u_{i},v_{i},t_{i}\right)={\left[X^{T}W(u_{i},v_{i},t_{i})X\right]}^{-1}X^{T}W(u_{i},v_{i},t_{i})Y$$

(3) $$X=\left(\begin{array}{cccc} 1 & x_{11} & \cdots & x_{1d}\\ 1 & x_{21} & \cdots & x_{2d}\\ \vdots & \vdots & \vdots & \vdots \\ 1 & x_{n1} & \cdots & x_{nd} \end{array}\right),Y=\left(\begin{array}{c} y_{1}\\ y_{2}\\ \vdots \\ y_{n} \end{array}\right)$$

where *X* is a matrix of influencing factors in observation *i* for separate space and time, *Y* is vector for HFRS cases in observation location *i*. *W*(*u_i_*,*v_i_*,*t_i_*) is an n × n spatial and temporal diagonal matrix. Its diagonal elements demonstrate the spatial and temporal weighting of influencing factors for observed location *i*. Deduced from Equation (2), *w_ij_*(*j* = 1,2,…,n) is named the spatial weight function. It can indicate the weight of another observation except for the observed location (*u_i_*,*v_i_*,*t_i_*) [21]. *W*(*u_i_*,*v_i_*,*t_i_*) is a calculated value based on each spatial and temporal point parameter. It can be expressed using Equation (4):

(4) $$W(u_{i},v_{i},t_{i})=diag(W_{1}(u_{i},v_{i},t_{i}),W_{2}(u_{i},v_{i},t_{i}),\cdots ,W_{n}(u_{i},v_{i},t_{i}))$$

When *W*(*u_i_*,*v_i_*,*t_i_*) is calculated, $\hat{\beta }\left(u_{i},v_{i},t_{i}\right)$ can be obtained according to Equation (2). HFRS cases variable *Y* at point (*u_i_*,*v_i_*,*t_i_*) is expressed as:

(5) $$\hat{y}(u_{i},v_{i},t_{i})=x_{i}^{T}\hat{\beta }(u_{i},v_{i},t_{i})$$

where $x_{i}^{T}=\left(1,x_{i1},x_{i2},\ldots ,x_{id}\right)$ represents the values of influencing factors at observed location (*u_i_*,*v_i_*,*t_i_*).

### 2.2. Spatial Weight Function Selection for SD-GTWR

Concluded from Tobler’s first law of geography “Everything is related to everything else, but near things are more related than distant things” [27], the correlation coefficient of HFRS cases between different counties is negatively associated with spatial distance. In order to clarify the autocorrelation of HFRS infection, effects from neighbors should be considered according to its spatial distance from the focal location [28].

In previous studies, results of the calculation should be directly influenced by the spatial weight function [21,22]. In recent research, the major functions for GWR-based models (GWR model and GTWR model) are the tri-cube kernel function, Gauss kernel function and bi-square kernel function [3,28]. In the tri-cube kernel function process, the situation for infinity in the regression point might happen. Gauss kernel function considers all observation data (such as observation points weak/none relevant to the current observation data) in data estimation. The bi-square kernel function excludes the unnecessary influences to eliminate the weak referenced observation points. It can be expressed as:

(6) $$w_{ij}=\{\begin{array}{ll} {\left[1-{\left(\frac{d_{ij}}{h}\right)}^{2}\right]}^{2} & d_{ij}\leq h\\ 0 & d_{ij}>h \end{array}$$

(7) $$d_{ij}=\sqrt{\lambda \left[{\left(u_{i}-u_{j}\right)}^{2}+{\left(v_{i}-v_{j}\right)}^{2}\right]+\mu {\left(t_{i}-t_{j}\right)}^{2}}$$

In the GWR model, *d_ij_* is the spatial distance between regression point (*u_i_*,*v_i_*) and (*u_j_*,*v_j_*). In the GTWR model, *d_ij_* is the spatial and temporal distance between regression point (*u_i_*,*v_i_*,*t_i_*) and another regression point (*u_j_*,*v_j_*,*t_j_*). *h* is not a negative value and represents the bandwidth. *h* indicates the relationship between *w_ij_* and *d_ij_*. 

In Equation (7), *λ* is spatial parameter to measure spatial distance. *μ* is a temporal parameter to measure temporal distance. In Equation (6), when the spatial and temporal weight function is ascertained, the bandwidth value *h* will directly impact the regression result. The reason is that *h* directly decides the value of *w_ij_* for point (*u_i_*,*v_i_*,*t_i_*). The selection of bandwidth value can be accomplished by using Cross Validation (CV), Generalized Cross Validation (GCV), the Akaike information criterion (AIC), Bias information criterion (BIC), etc.

Using each approach, the operation time will increase exponentially with the growing size of the statistical data. To solve this problem, Incremental Spatial Autocorrelation (ISA) is utilized to specify an initial bandwidth value. As ISA method sharply narrows the selection range of bandwidth, correspondingly reducing the operation time to a certain degree.

### 2.3. SD-GTWR

Research has demonstrated that HFRS happens as a characteristic of seasonal or cyclic time series [3,11]. In 1976, ARIMA was developed by Box and Jenkins to forecast non-seasonal time series. To deal with seasonal data like HFRS cases, Box and Jenkins developed an extension for ARIMA model called Seasonal Autoregressive Integrated Moving Average (S-AIMA). It uses a seasonal difference method to obtain stabilized data. Seasonal differences are used to deal with the non-stationary time series.

Following the research on ARIMA and S-ARIMA done by Box and Jenkins, the SD-GTWR model was constructed. Time series analysis and autocorrelation analysis were conducted to ensure the feasibility of using seasonal difference methods. The GTWR model is a prerequisite for doing an advanced seasonal difference model to use seasonal differences. In our previous research, HFRS cases in Hubei Province displayed a bimodal seasonal distribution pattern rather than a linear distribution during 1980–2000 [6]. Seasonal differences calculation (SD-GTWR) for HFRS cases in Hubei Province can be expressed as Equation (8) based on the GTWR model:

(8) $$W_{t}=X_{t}-X_{t-s}$$

*W_t_* is the result of seasonal difference on time *t*. S means the time periods of seasonal difference. *X_t_* represents the HFRS cases vector in time *t*. *X_t−s_* represents the HFRS cases vector value S units before time *t*. Different from GTWR, vector *W_t_* is used as a dependent variable to progress the procedures instead of the initial cases data.

## 3. Implementation of SD-GTWR for HFRS

A series of tests were conducted to identify the spatial and temporal characteristics of the HFRS epidemic. First, it should be verified whether the HFRS infection data demonstrates a spatial autocorrelation feature. Secondly, time sequence analysis should be adopted to verify the seasonal characteristics for HFRS. The first and second steps should provide a seasonal difference characteristics result for HFRS. Thirdly, OLS, GWR-based model (including GWR, GTWR) and SD-GTWR should be performed respectively based on the HFRS infections data. The purpose of this implementation was to improve the reliability and accuracy of the SD-GTWR model.

### 3.1. Study Data

The study area is Hubei Province, which is located in the south-central part of China. With an area of 186,000 square kilometers, it lies in the middle reaches of the Yangtze River. It is situated at 108’21”–116’07” east longitude and 29’05”–33’20” north latitude. Jianghan Plain takes up most of the central and southern area, while mountains are found in the west area. Hubei Province has thousands of square kilometers of plains area, and also possesses large areas of hills and mountainous regions. In its total area, mountainous regions account for 56% and hills occupy 24%, while the remaining 20% being water area. The two major rivers, the Yangtze River and Hanshui tributary flow through it. The Yangtze river is 1061 km in length in the Hubei Province and occupies about 1416 km^2^ of drainage area. The Hanshui tributary is 878 km in length and occupies about 450 km^2^ of drainage area. There are thousands of lakes in Jianghan Plain, the largest two being Liangzi Lake and Hong Lake. 

The study data covers the period from 2011 to 2015. Basic geographic information data of Hubei Province was collected from the Chinese National Administrator of Surveying, Mapping and Geo-Information. The geographic data contains the counties’ administrative regions by name and code. HFRS case data and rodent density data were provided by Hubei Provincial Center for Disease Control and Prevention and Chinese Center for Disease Control and Prevention. HFRS case data contains the monthly case values for each county. Climate data was obtained from the National Centers for Environmental Prediction and Hubei Meteorological Bureau. Climate data contains monthly average temperature, humidity and rainfall for each county. Human population density data, which includes the annual population for each county, was extracted from the Hubei Statistical Yearbook.

HFRS in China was mainly caused by two types of hantavirus (HTNV transmitted by *Apodemus agrarius* and SEOV transmitted by *Rattus norvegicus*) [5,14,29]. In Hubei Province, HFRS was mainly in the form of SEOV [6]. There are many factors that should be considered when performing a regression analysis for HFRS [24,30,31]. Climate influencing factors were commonly adopted. In this study, temperature, rainfall, relative humidity, SOI and air pressure factors were analyzed as the climate factors for HFRS in Yingshang County [14]. Eco-geographical factors such as intensity of human activity, climate conditions, and landscape elements have been proven to affect the occurrence of HFRS in Changsha, Hunan Province. Derived from the previous research, average temperature, average humidity, average rainfall, human population density, rodent population density, water area and county average surface elevation were included to execute our model.

### 3.2. Temporal and Spatial Non-Stationary Diagnosis

**Figure 1:**
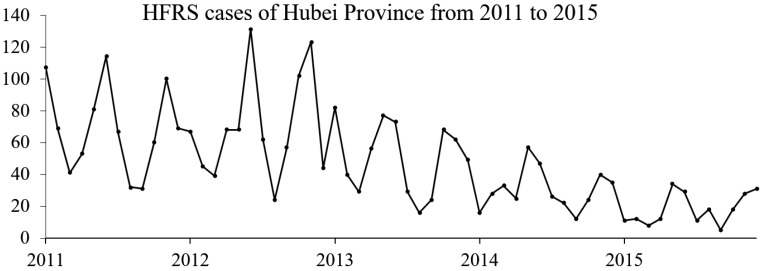
HFRS cases of Hubei Province from 2011 to 2015.

Figure 1 demonstrates the time series for HFRS cases in Hubei Province from 2011 to 2015. It indicates that the number of HFRS cases fluctuated during this period. In general, the number of case is decreased over the time. A nonlinear distribution pattern appears from the curve. This finding reveals that HFRS cases data cannot be stationary distributed temporally.

**Figure 2:**
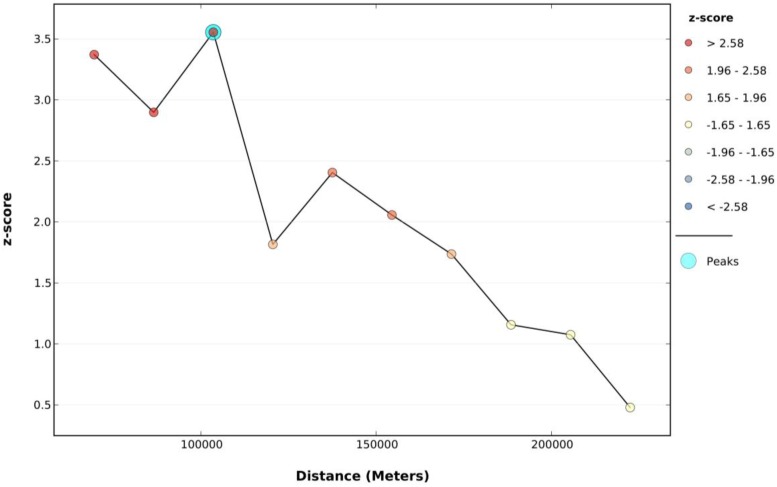
Incremental Spatial Autocorrelation (ISA) analysis results.

Incremental Spatial Autocorrelation (ISA) was provided by the ArcGIS 10.2 software (PESRI, Redlands, CA, USA), which used Spatial Autocorrelation (Global Moran’s I) tool for a series of increasing distances. ISA also measured the intensity of spatial clustering for each distance. Table 1 and Figure 2 displays Moran’s Index value in different distances provided by Incremental Autocorrelation Analysis tool. Z-scores reflect the intensity of spatial clustering. A statistically significant peak Z-score indicates distances in which spatial clustering is the most prominent. A Z-score peak represents the best fixed distance value in spatial regression analysis. In this study, a Z-score peak emerged when the distance was set to 103,540.34. The initial calculation bandwidth for GWR and GTWR model was set to 103,540.34. 

### 3.3. Parameter Selection

After defining a bandwidth value, it is important to define the spatial and temporal balancing parameters *λ* and *μ*. In the GTWR model, the range of geo-location coordinates and date time are on totally different scales, necessitating the two parameters be set into a unified range scale for computation. Based on difference reciprocal, *λ* value was defined as the maximum spatial value, *μ* value was defined as the minimum temporal value. The units of space and time were set as km and month, respectively. In Hubei Province, the maximum distance between two neighboring counties is about 800 km. The sample time frame is 60 months (12 months per year multiplied by 5 years). According to reciprocal weight values, *λ* was set to 3, *μ* was set to 40.

### 3.4. Seasonal Analysis

**Figure 3:**
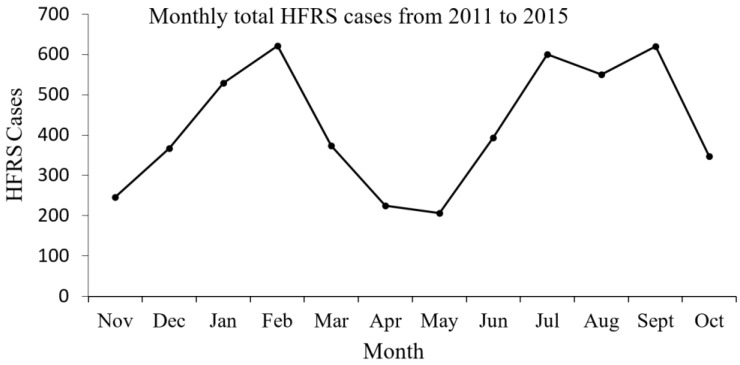
Monthly total HFRS cases from 2011 to 2015.

Figure 3 reveals the overall monthly distribution pattern of HFRS in Hubei Province from 2011 to 2015. In Figure 3, HFRS epidemics are bimodally distributed, and peaks appear on February and August. The seasonal difference range indicates the linear relationships among future values, current values and past values.

Hubei Province has four distinctly different seasons. The climate of each season varies largely. Previous studies have demonstrated that outbreaks of HFRS epidemics were strongly related with climate influencing factors [6], and the HFRS cases in Hubei Province displayed a bimodal distribution for each year [32,33]. From Figure 1 and Figure 3, it also appears that two peaks happen in a year (12 months). Accordingly, it seems that it is better to narrow down the seasonal difference range for each year to 6 months [34]. As a result of the above reasons, the time frame for the seasonal difference calculations was set to 6 months.

After determining the seasonal difference value, it is inevitable to set up independent and dependent values. In our research data, there were 5 (years) × 12 (months) × 76 (counties) rows. Independent variables were set as a (4560 × 7) matrix, expressed as X. The dependent variable was set as a (4560 × 1) matrix, expressed as Y. HFRS cases data was seasonally different with a six month interval (peaks happened in February and August). The seasonally different dependent variable matrix can be expressed as YDif.

## 4. Results and Discussion

### 4.1. Correlation Analysis on Influencing Factors

Correlation analysis was used to analyze the correlation of the influencing factors, including average temperature (Avertemp), average humidity (Averhumi), average rainfall (Rainacc), Area, rodent density (RodentDensity), human population density (PopDensity), water area (WaterArea) and surface mean elevation (MeanHeight). In Table 2, the 5% significant level is marked as “*”, while “**” represents a 1% significance level. The result shows that five factors are statistically significant (*p* < 0.10), which are rodent density (Rodent Density), human population density (PopDensity), water area (Water Area), average temperature (Avertemp) and average surface elevation (MeanHeight). The following regression analysis should select the five factors as influencing factors. 

### 4.2. Compared with OLS Model

Table 3 reflects the model diagnostics result. The R square value is 0.447, which means that 44.7% of the variation for HFRS cases and possible influencing factors can be explained. 

Table 4 expresses the parameter estimation results of the OLS model. Average temperature, average humidity, rodent population density, human population density and mean height have significant correlations with HFRS incidents. In Table 4, parameter B represents the regression intercept for parameters [35]. The values of B indicate that the independent variables are associated, either positively or negatively. It can be inferred from Table 4 that factors including average humidity (Averhumi), rodent density (RodentDensity) and human population density (PopDensity) are positively associated with the infection of HFRS. Factors including average temperature (Avertemp) and average surface elevation (MeanHeight) are negatively associated with HFRS cases.

### 4.3. Compared Results among GWR-Based Models

Regarding the test on goodness of fit, Table 5 indicates that R square for the GWR model is 0.54. This value is higher than the value from the OLS model in Table 3 (0.447). This means that in terms of fitting the data, the GWR model that incorporated the temporal and seasonal effects achieves a 22.7% improvement as compared to the OLS model.

Along with the R square value, the corrected Akaike Information Criterion (AICc) also measures the regression level for models. AICc is not an absolute measure of goodness of fit, however, it is useful for test models with different explanatory variables when applying the same dependent variable [36,37]. The lower AICc value a model has, the better fitness the observed data provides. As presented in Table 5, R square values for GWR, GTWR and SD-GTWR are 0.54, 0.61 and 0.77. The corresponding AICc values are 3539.23, 3462.41, 3421.06. Therefore, we can infer from the statistical results that the GTWR model is better than the GWR model by 13.0% and that the SD-GTWR model is better than the GTWR model by 26.2%. There could be two reasons for this phenomenon. First, in GTWR-based models (GTWR and SD-GTWR), the temporal factors covered more information than they did in the GWR model. Secondly, considering the seasonal pattern of HFRS cases, the SD-GTWR model simulated the relationships between HFRS epidemic and the possible influencing factors much better [37].

Table 6 shows the F-tests results for the three models and their corresponding *p*-values. Variables reach 5% level significant are marked with “*”. It can be inferred that different significant variables can be found when using different models. In the GWR model, only climate factors including average temperature (Avetemp), average humidity (Averhumi) and average rainfall (Rainacc) are considered statistically significantly influencing factors. In the GTWR model, including the climate factors (as the GWR model discovered), rodent density (RodentDensity) and surface mean elevation (MeanHeight) are also regarded as influencing factors. In the SD-GTWR model, the water area (WaterArea) and human population density (PopDensity) factors are also selected for the estimation of HFRS cases. On the other hand, average temperature factor is excluded from the SD-GTWR model.

The GWR model adds spatial characteristics to the general linear regression when processing the data. It is a non-stationary regression method. The GWR model is much better than OLS model in simulating the HFRS trends. First, HFRS demonstrates a strong spatial and temporal autocorrelation characteristic [37]. Neglecting its spatial variation may have a great impact on any simulation results. Secondly, HFRS from neighboring regions could contribute to the emergence of HFRS in the focal county. This phenomenon can be ascribed to the global and local autocorrelation characteristics of HFRS in Hubei Province.

Compared with the GWR model, R-square values were much higher in the GTWR and SD-GTWR models. Regression parameters were utilized as functions to describe the spatial and temporal position of sample data in the GTWR-based models (the GTWR model and SD-GTWR model). The calculation accuracy of the two models are higher than that of the GWR model, because that spatial and temporal weights in these functions can better respond to the influencing factors in different spatial and temporal locations.

## 5. Conclusions

Regression models were commonly used to evaluate the relationships between possible influencing factors and the number of HFRS epidemic cases. The GTWR model was used to speculate about the connections between influencing factors and the number of HFRS epidemics. With the consideration of spatial and temporal variation, simulation results from the GTWR model were more accurate than those from non-spatial models like OLS or from a non-temporal model like GWR. Combined with the seasonal characteristics of the HFRS epidemic in Hubei Province, a new model named SD-GTWR was initially developed to conduct regression analysis on HFRS cases. Estimations have been made by different models such as OLS, GWR, GTWR and SD-GTWR. It can be inferred from the model diagnosis results that with the process of seasonal difference, the SD-GTWR model better simulated the correlations of the possible influencing factors.

The regression results from different models revealed the characteristics of different influencing factors in Hubei Province in 2011–2015. The following conclusions can be reached: 

First, the models applied in this paper demonstrated the relationships between HFRS epidemics and meteorological factors at different levels. Meteorological factors notably impacted the changing trends of HFRS outbreaks, for the reason that they are associated with the spatial presence of this infection.

Secondly, one of the influencing factors, average humidity, has been demonstrated as significantly associated with the HFRS outbreaks in the GWR, GTWR and SD-GTWR models. It can be interpreted that this factor might have a strong impact on the HFRS epidemic in Hubei Province. 

Thirdly, different models showed different estimation parameter results. This is reasonable since the OLS model does not consider spatial heterogeneity, which means that coefficients with spatial variation characteristics like rainfall may not be obvious. Moreover, the spatial and temporal variation of rodent density and surface mean elevation share the same distributions. It can be inferred that the model estimation results are dominantly affected by the spatial and temporal variation of HFRS. 

## Figures and Tables

**Table 1:** Global Moran's I Summary by Distance.

Distance (m)	Moran‘s Index	Expected Index	Variance	z-Score	*p*-Value
69,575.78	0.231009	−0.013333	0.005253	3.371300	0.000748
86,558.06	0.156391	−0.013333	0.003429	2.898408	0.003751
103,540.34	0.154798	−0.013333	0.002237	3.554875	0.000378
120,522.62	0.060104	−0.013333	0.001635	1.816059	0.069361
137,504.90	0.070680	−0.013333	0.001221	2.404742	0.016184
154,487.17	0.050333	−0.013333	0.000958	2.057034	0.039683
171,469.45	0.034890	−0.013333	0.000771	1.736562	0.082465
188,451.73	0.015889	−0.013333	0.000638	1.157021	0.247264
205,434.01	0.011405	−0.013333	0.000529	1.075416	0.282188
222,416.29	−0.003347	−0.013333	0.000435	0.479006	0.631935

**Table 2:** Correlation analysis results.

Factors	Correlation Coefficient	Significance (2-Tailed)
Avertemp	0.291 **	0.000
Averhumi	0.065	0.408
Rainacc	0.127	0.110
Area	−0.085	0.285
RodentDensity	0.223 **	0.009
PopDensity	0.372 **	0.000
WaterArea	0.352 **	0.000
MeanHeight	−0.416 **	0.000

** Correlation is significant at the 0.01 level (2-tailed); * Correlation is significant at the 0.05 level (2-tailed).

**Table 3:** OLS model summary.

R	R Square	Adjusted R Square	Std. Error of the Estimate
0.668 ^a^	0.447	0.381	0.751

^a^ Predictors: (Constant), Rainacc, PopDensity, WaterArea, RodentDensity, Averhumi, Area, MeanHeight, Avertemp.

**Table 4:** OLS coefficient diagnosis.

Variables	Unstandardized Coefficients		Standardized Coefficients	t	Significance	95.0% Confidence Interval for B	
	B	Std. Error	Beta			Lower Bound	Upper Bound
(Constant)	−1.816	4.836			0.708	−11.469	7.836
Avertemp	−0.011	0.016	−0.129	−0.736	0.046 **	−0.042	0.020
Averhumi	0.045	0.046	0.124	0.970	0.003 **	−0.047	0.137
Rainacc	0.001	0.001	0.096	0.647	0.520	−0.001	0.002
Area	1.357 × 10^−8^	0.000	0.238	1.942	0.056	0.000	0.000
RodentDensity	0.223	0.210	0.110	1.062	0.002 **	−0.196	0.641
PopDensity	9.685 × 10^−7^	0.000	0.072	0.710	0.004 **	0.000	0.000
WaterArea	0.000	0.001	−0.060	−0.530	0.598	−0.002	0.001
MeanHeight	−0.002	0.000	−0.795	−5.119	0.000 **	−0.003	−0.001

** Correlation is significant at the 0.01 level (2-tailed).

**Table 5:** GWR-based model summary.

Diagnostic Information	GWR	GTWR	SD-GTWR
Residual sum of squares	2246.65	2091.89	1410.61
Classic AIC	3530.59	3455.22	3336.86
AICc	3539.23	3462.41	3421.06
BIC/MDL	3820.08	3719.96	3181.89
CV	2.84	2.73	2.42
R square	0.54	0.61	0.77
Adjusted R square	0.43	0.49	0.67

**Table 6:** GWR non-stationary of parameters for the GWR, GTWR and SD-GTWR models.

Parameter	GWR		GTWR		SD-GTWR	
	F	*p*-Value	F	*p*-Value	F	*p*-Value
Avertemp	**10.1175**	0.0245 *	**15.2382**	0.0114 *	5.1334	0.0728
Averhumi	**9.0022**	0.0301 *	**9.7389**	0.0262 *	**7.5971**	0.0400 *
Rainacc	**8.5417**	0.0329 *	**6.7935**	0.0479 *	**6.9314**	0.0464 *
Area	0.9766	0.3684	3.6008	0.1162	4.6261	0.0842
RodentDensity	0.2116	0.6649	**7.3263**	0.0424 *	**22.1009**	0.0053 *
PopDensity	0.6296	0.4635	3.0188	0.1428	**19.4055**	0.0070 *
WaterArea	1.7109	0.2478	0.5724	0.4834	**19.9125**	0.0066 *
MeanHeight	2.3200	0.1882	**10.6680**	0.0223 *	**25.297**	0.0040 *

Bold font stand for variables are significant at the 0.05 level. * Correlation is significant at the 0.05 level (2-tailed).
